# Identification of a Chemoreceptor in *Pseudomonas aeruginosa* That Specifically Mediates Chemotaxis Toward α-Ketoglutarate

**DOI:** 10.3389/fmicb.2016.01937

**Published:** 2016-11-29

**Authors:** David Martín-Mora, Alvaro Ortega, José A. Reyes-Darias, Vanina García, Diana López-Farfán, Miguel A. Matilla, Tino Krell

**Affiliations:** Department of Environmental Protection, Estación Experimental del Zaidín, Consejo Superior de Investigaciones CientíficasGranada, Spain

**Keywords:** chemotaxis, chemoreceptor, signal transduction, molecular recognition, *Pseudomonas aeruginosa*, α-ketoglutarate

## Abstract

*Pseudomonas aeruginosa* is an ubiquitous pathogen able to infect humans, animals, and plants. Chemotaxis was found to be associated with the virulence of this and other pathogens. Although established as a model for chemotaxis research, the majority of the 26 *P. aeruginosa* chemoreceptors remain functionally un-annotated. We report here the identification of PA5072 (named McpK) as chemoreceptor for α-ketoglutarate (αKG). High-throughput thermal shift assays and isothermal titration calorimetry studies (ITC) of the recombinant McpK ligand binding domain (LBD) showed that it recognizes exclusively α-ketoglutarate. The ITC analysis indicated that the ligand bound with positive cooperativity (*K*_d1_ = 301 μM, *K*_d2_ = 81 μM). McpK is predicted to possess a helical bimodular (HBM) type of LBD and this and other studies suggest that this domain type may be associated with the recognition of organic acids. Analytical ultracentrifugation (AUC) studies revealed that McpK-LBD is present in monomer-dimer equilibrium. Alpha-KG binding stabilized the dimer and dimer self-dissociation constants of 55 μM and 5.9 μM were derived for ligand-free and αKG-bound forms of McpK-LBD, respectively. Ligand-induced LBD dimer stabilization has been observed for other HBM domain containing receptors and may correspond to a general mechanism of this protein family. Quantitative capillary chemotaxis assays demonstrated that *P. aeruginosa* showed chemotaxis to a broad range of αKG concentrations with maximal responses at 500 μM. Deletion of the *mcpK* gene reduced chemotaxis over the entire concentration range to close to background levels and wild type like chemotaxis was recovered following complementation. Real-time PCR studies indicated that the presence of αKG does not modulate *mcpK* expression. Since αKG is present in plant root exudates it was investigated whether the deletion of *mcpK* altered maize root colonization. However, no significant changes with respect to the wild type strain were observed. The existence of a chemoreceptor specific for αKG may be due to its central metabolic role as well as to its function as signaling molecule. This work expands the range of known chemoreceptor types and underlines the important physiological role of chemotaxis toward tricarboxylic acid cycle intermediates.

## Introduction

Bacteria possess a variety of signal transduction systems that allow them to adapt their metabolism and behavior to different changes in environmental cues. One of the major signal transduction mechanisms is based on the action of chemosensory signaling pathways (Wuichet and Zhulin, 2010). Typically, signaling is initiated by the binding of signals to the chemoreceptor ligand binding domain (LBD), which in turn triggers a molecular stimulus that modulates autophosphorylation of the CheA histidine kinase and consequently transphosphorylation of the CheY response regulator (Hazelbauer et al., 2008). Chemosensory systems carry out multiple functions such as mediating chemotaxis, type IV pili-based motility or alternative cellular processes (Hickman et al., 2005; Zusman et al., 2007; Wuichet and Zhulin, 2010).

*Escherichia coli* is the traditional model to study chemoreceptor-based signaling processes (Parkinson et al., 2015). This bacterium has 5 chemoreceptors, of which 4 contain a periplasmic 4-helix bundle LBD. The fifth receptor, Aer, causes aerotaxis and has a cytosolic PAS type LBD. Signals bind either directly to the LBD or in complex with a periplasmic ligand binding protein. Chemoreceptors feed into a single chemosensory cascade that mediates chemotaxis toward compounds like sugars, amino acids or dipeptides.

The analysis of 450 bacterial genomes showed that approximately half of them possess genes encoding chemosensory signaling proteins (Wuichet and Zhulin, 2010). Frequently, chemosensory signaling is in many bacteria more complex than in *E. coli*. Bacteria that possess chemosensory signaling proteins have on average 14 chemoreceptor genes (Lacal et al., 2010b) and for some species up to 60 chemoreceptors were identified (Matsunaga et al., 2005). Genome analyses have furthermore demonstrated that other bacteria possess different types of chemoreceptors that differ in the type of LBD. Most chemoreceptors are functionally un-annotated but such knowledge is indispensable to identify the forces that have shaped the evolution of chemotactic behavior. Chemoreceptors can be classified according to the size of their LBD into cluster I (∼150 amino acids) or cluster II (∼250) (Lacal et al., 2010b). Cluster II receptors are absent from *E. coli* but were estimated to correspond to 40% of all chemoreceptors (Lacal et al., 2010b). The main representatives of cluster II LBDs are the dCACHE (Liu et al., 2015; Upadhyay et al., 2016) and helical bimodular domain (HBM) (Pineda-Molina et al., 2012; Ortega and Krell, 2014) which, despite their abundance, remain poorly characterized.

The first receptor characterized with an HBM domain was McpS of *P. putida* KT2440 that mediated chemotactic responses to different Krebs cycle intermediates (Lacal et al., 2010a, 2011a). The 3D structure of the cluster II LBD of McpS revealed that it corresponds to a novel bacterial sensor domain composed of two structural modules that each can bind directly signal molecules (Pineda-Molina et al., 2012). Other HBM domain containing receptors are the citrate specific McpQ of *P. putida* KT2440 (Martin-Mora et al., 2016) as well as McfS (Parales et al., 2013) of *P. putida* F1 and McpS of *P. fluorescens* Pf0-1 (Oku et al., 2014) that mediate chemotaxis to organic acids. HBM domains were found to form part of chemoreceptors and sensor kinases and were found in bacteria and archaea (Ortega and Krell, 2014).

*Pseudomonas aeruginosa* is a ubiquitously occurring microorganism that is capable of causing multiple human opportunistic infections (Gellatly and Hancock, 2013). As such, *P. aeruginosa* is the leading cause of nosocomial infections, particularly in immunocompromised, cancer, burn and cystic fibrosis patients (Juhas, 2015). This, combined with the emergence of strains resistant to all commercially available antibiotics, makes *P. aeruginosa* one of the most feared pathogens (Dorotkiewicz-Jach et al., 2015). In addition, *P. aeruginosa* was found to colonize (Walker et al., 2004) and infect different plants (Cao et al., 2001). A number of reports show that *P. aeruginosa* chemotaxis is necessary for efficient host colonization and virulence (Garvis et al., 2009; McLaughlin et al., 2012; Kamath et al., 2016; Schwarzer et al., 2016) and the interference with the motility and chemotaxis was proposed as an alternative strategy to block this pathogen (Erhardt, 2016).

*Pseudomonas aeruginosa* is a model organism to study chemotaxis (Kato et al., 2008; Sampedro et al., 2015). In particular its responses to amino acids by the three paralogous receptors PctA, PctB, and PctC (Kuroda et al., 1995; Taguchi et al., 1997; Rico-Jimenez et al., 2013; McKellar et al., 2015; Reyes-Darias et al., 2015a,b) as well as its response to inorganic phosphate (Wu et al., 2000; Rico-Jimenez et al., 2016) by the CtpL and CtpH receptors have been studied in some depth. In addition, the cytosolic and atypical receptor McpB (also named Aer2) (Watts et al., 2011; Airola et al., 2013; Garcia-Fontana et al., 2014; Garcia et al., 2016) was subject to many studies and is amongst the best studied members of the chemoreceptor sub-family with cytoplasmic location. Chemoreceptor PA2652 was identified as a specific malate chemoreceptor (Alvarez-Ortega and Harwood, 2007) and TlpQ responsible for the chemotaxis toward the plant hormone ethylene (Kim et al., 2007). Two other chemoreceptors, WspA and BdlA, play important roles in biofilm formation and dispersion (Hickman et al., 2005; Morgan et al., 2006; O’Connor et al., 2012; Petrova and Sauer, 2012). However, of the 26 *P. aeruginosa* chemoreceptors more than half remain of unknown function. This knowledge, however, is indispensable to understand the forces that have driven the evolution of chemotaxis in this ubiquitous pathogen.

*Pseudomonas aeruginosa* PAO1 has three receptors with an HBM domain. One of them, CtpL, was found to mediate specifically chemotaxis to low Pi concentrations (Wu et al., 2000). However, CtpL does not bind Pi directly but recognizes the Pi loaded periplasmic binding protein PstS (Rico-Jimenez et al., 2016). The remaining two receptors, PA1646 and PA5072, are of unknown function. We report here the functional annotation of one of them, PA5072, that binds and mediates chemotaxis exclusively to α-ketoglutarate (αKG). This receptor, termed McpK, expands the range of known chemoreceptor types.

## Materials and Methods

### Bacterial Strains, Plasmids and Primers

The strains and plasmids used in this study are listed in **Table 1**. Different primers for molecular biology manipulations are provided in **Supplementary Table S1**.

**Table 1:** Bacterial strains and plasmids used in this study.

Strain or plasmid	Relevant characteristics	Reference or source
**Strains**		
*Escherichia coli* BL21 (DE3)	F-, *ompI, hsdS*_B_ (r^-^_B_ m^-^_B_) *gal*, *dam*, *met*	Jeong et al., 2009
*E. coli* DH5α	*supE44 lacU169* (*∅80lacZ*ΔM15) *hsdR17* (*r_k_*^-^*m_k_*^-^), *recA1 endA1 gyrA96 thi-1 relA1*	Woodcock et al., 1989
*E. coli* HB101	F- Δ*(gpt-proA)62 leuB6 supE44 ara-14 galK2 lacY1*Δ*(mcrC-mrr) rpsL20* (Sm^r^) *xyl-5 mtl-1 recA13 thi-1*	Boyer and Roulland-Dussoix, 1969;Kessler et al., 1992
*E. coli* CC118λ*pir*	Rif^r^; Δ(*ara-leu*) *araD*Δ*lacX74 galE galK phoA20 thi-1 rpsE rpoB argE*(Am) *recA1* Tn*7* λpir	Herrero et al., 1990
*Pseudomonas aeruginosa* PAO1	Reference strain	Stover et al., 2000
*P. aeruginosa* PAO1-Km	Km^r^; wild type PAO1 with a Km cassette inserted in a neutral position downstream of *glmS*	This study
*P. aeruginosa* PAO1 Δ*mcp*K	*P. aeruginosa* PAO1 deletion mutant of *ΔmcpK* gene	This study
**Plasmids**		
pGEM-T	Ap^r^; TA-cloning vector	Promega
pET28b(+)	Km^r^; Protein expression plasmid	Novagen
p34S-Km3	Km^r^, Ap^r^; Km3 antibiotic cassette	Dennis and Zylstra, 1998
pET28-McpK-LBD	Km^r^; pET28b(+) derivative containing DNA fragment encoding McpK-LBD	This study
pUC18NotI	Ap^r^; *ori* pMB1, similar to pUC18 but with NotI sites flanking the MCS; cloning vector	Purchased from Biomedal
pUC18NotI-5072Up	Ap^r^; pUC18NotI derivative containing a 0.3-kb HindIII-XbaI fragment of upsteam region of *pa5072*	This study
pMAMV257	Ap^r^; 1.4-kb PCR product containing the intergenic region between genes *pa5548* and *pa5549* (*glmS*) of *P. aeruginosa* PAO1 was inserted into the EcoRI/HindIII sites of pUC18NotI. A BamHI site was inserted into this intergenic region by PCR.	This study
pMAMV258	Ap^r^, Km^r^; 0.96-kb BamHI fragment containing *km3* cassette of p34S-Km3 was inserted into the BamHI downstream of *glmS* in pMAMV257	This study
pUC18NotI-5072UpDw	Ap^r^; pUC18NotI derivative containing a 1-kb HindIII-EcoRI fragment containing upstream and downstream region of *pa5072*	This study
pKNG101	Sm^r;^ *ori*R6K, *mob, sac*; suicide vector	Kaniga et al., 1991
pKNG101-PA5072UpDw	Sm^r^; pKNG101derivative containing a 0.6 Kb NotI fragment from pUC18NotI-5072UpDw cloned into pKNG101	This study
pMAMV261	Sm^r^, Km^r^; 2.4-kb NotI fragment of pMAMV258 was cloned at the same site in pKNG101	This study
pRK600	Cm^r^; *ori*ColE1, RK2 *mob^+^, tra^+^*; helper plasmid	de Lorenzo et al., 1990
pBBR1MCS-2	Km^r^; broad-host-range cloning plasmid pBB1MCS derivative containing Km^r^ cassette, *mob^+^, rep^+^*	Kovach et al., 1995
pBBR1MCS-2-McpK	Km^r^; pBBR1MCS-2 derivative containing *mcpK* gene and its 607 bp upstream region	This study


*The following abbreviations were used for antibiotics: km, kanamycin; cm, chloramphenicol; ap, ampicillin; rif, rifampicin; sm, streptomycin.*

### Construction of Expression Plasmid for McpK-LBD

The DNA fragment of *mcpK* encoding amino acids Arg^38^–Ser^293^ was amplified using primers McpK-LBD_fw and McpK-LBD_rv and genomic DNA of *P. aeruginosa* PAO1. The resulting PCR product was cloned into pGEM-T and digested with NdeI and BamHI and then subcloned into the expression plasmid pET28b(+) linearized with the same enzymes. The resulting plasmid, termed pET28-McpK-LBD, was verified by DNA sequencing of the insert and flanking regions.

### Overexpression and Purification of McpK-LBD

*Escherichia coli* BL21 (DE3) containing pET28-McpK-LBD was grown in 2 L Erlenmeyer flasks containing 500 ml LB medium supplemented with 50 μg ml^-1^ kanamycin at 30°C until an OD_660_ of 0.6, at which point protein production was induced by adding 0.1 mM isopropyl-β-D-1-thiogalactopyranoside (IPTG). Growth was continued at 18°C overnight before cell harvest by centrifugation at 10,000 *g* for 30 min. All subsequent manipulations were carried out at 4°C. Cell pellets were resuspended in buffer A [20 mM Tris/HCl, 200 mM NaCl, 10 mM imidazole, 5% (vol/vol) glycerol, pH 8.0] and broken by French press treatment at 1000 psi. After centrifugation at 20,000 *g* for 1 h, the supernatant was loaded onto a 5 ml HisTrap column (Amersham Bioscience), washed with five column volumes of buffer A and eluted with a 30–300 mM imidazole gradient in buffer A. Protein-containing fractions were pooled.

### Thermal Shift Assays

Thermal shift assays were performed using a BioRad MyIQ2 Real-Time PCR instrument. Ligands were prepared by dissolving Biolog Phenotype Microarray compounds in 50 μl of MilliQ water to obtain a final concentration of around 10–20 mM (as indicated by the manufacturer). Screening was performed with plates PM1, PM2A, PM3B, PM4A, and PM5. Each plate contains 95 compounds and a control (**Supplementary Figure S1**). Each 25 μl assay mixture contained 40 μM protein in 5 mM Tris, 5 mM Pipes, 5 mM Mes, pH8.0 and SYPRO orange (Life Technologies) at 5x concentration. Aliquots of 2.5 μl of the resuspended Biolog compounds were added to each well. Samples were heated from 23°C to 85°C at a scan rate of 1°C/min. The protein unfolding curves were monitored by detecting changes in SYPRO Orange fluorescence. Melting temperatures were determined using the first derivative values from the raw fluorescence data.

### Isothermal Titration Calorimetry

Experiments were conducted on a VP-microcalorimeter (Microcal, Amherst, MA, USA) at 20°C or 10°C. McpK-LBD was dialyzed overnight against 5 mM Tris, 5 mM PIPES, 5 mM MES, pH 8.0 and placed into the sample cell. Typically, protein at 20–100 μM was introduced into the sample cell and titrated with 1–3 mM ligand solutions that were prepared in dialysis buffer immediately before use. For fitting, data were integrated using NITPIC (Keller et al., 2012) before global fitting to a two symmetric-site binding model in SEDPHAT (Houtman et al., 2007). The binding constants expressed are corrected as *K*_d_1 = 2^∗^*K*_d_1′ and *K*_d_2 = 0.5^∗^*K*_d_2′ in order to express an estimation of the microscopic constants for the two binding sites model, where Kx’ are the macroscopic constants measured. The cooperativity factor α is expressed as α = *K*_d_1/*K*_d_2. Statistical uncertainties for best-fit estimates of *K*_d_ and Δ*H* were calculated using standard error surface projection methods built into SEDPHAT.

### Analytical Ultracentrifugation

Experiments were performed in a Beckman Coulter Optima XL-I analytical ultracentrifuge (Beckman-Coulter, Palo Alto, CA, USA) equipped with UV-visible absorbance as well as interference optics detection systems, using an An50Ti 8-hole rotor and 12 mm path-length charcoal-filled epon double-sector centerpieces. The experiments were carried out at 7°C with at least 1 h stabilizing after reaching 7°C in the rotor chamber, using 5 mM Tris, 5 mM PIPES, 5 mM MES, pH 8.0. Samples of 10–50 μM for McpK-LBD were analyzed in the presence and absence of 1 mM αKG.

Sedimentation velocity (SV) runs were carried out at a rotor speed of 128,793 × g using 400 μL samples with McpK-LBD dialysis buffer as reference. A series of 180 scans without time intervals between successive scans were acquired for each sample. Laser at a wavelength of 236 nm was used in the absorbance optics mode. A least squares boundary modeling of the SV data was used to calculate sedimentation coefficient distributions with the size-distribution *c(s)* method and the non-interacting discrete species model (Schuck, 2000) implemented in the SEDFIT v14.1 software. The molecular weight was extracted from the sedimentation profiles, via the Lamm equation solution included in the *c(s)* model of SEDFIT (Schuck, 2000). The best fit values obtained for the sedimentation coefficient (*s*, in S or Svedbergs) and diffusion were used to estimate the molar mass of the molecule using the Svedberg equation. Buffer density (*ρ* = 1.00036 g/ml) and viscosity (*η* = 0.01433 Poise) at 7°C were estimated by SEDNTERP software (Laue et al., 1992) from the buffer components. The partial specific volume used was 0.71274 ml/g as calculated from the amino acid sequence also using SEDNTERP software.

Sedimentation equilibrium (SE) experiments were performed at 7°C, measuring absorbance at 280 nm as a function of radius. Three different concentrations of McpK-LBD (40, 50, and 60 μM) both in the absence or in the presence of 1 mM αKG were loaded with the dialysis buffer as reference and a multi-speed (9,740, 20,606, and 185,462 × g) run was used. The data were analyzed globally by the SEDPHAT (Vistica et al., 2004) “species analysis with mass conservation constraints” model. The goodness of fit was evaluated on the basis of the residuals, expressed as the difference between the experimental data and the theoretical curve.

### Construction of *P. aeruginosa* PAO1 Δpa5072 (ΔmcpK)

An unmarked non-polar deletion of *pa5072* was created by allelic exchange as described (Schweizer, 1992) with the following modifications: two DNA fragments comprising the 234 bp upstream and 336 bp downstream regions of *pp5072* were obtained by PCR amplification of genomic DNA using primers PA5072UpF-HindIII and PA5072UpR-XbaI (upstream region) and primers PA5072DownF-XbaI and PA5072DownR-EcoRI (downstream region). The upstream DNA fragment was digested with HindIII and XbaI and cloned into pUC18NotI digested with the same restriction enzymes, resulting in pUC18NotI-5072Up, whereas the downstream DNA fragment was digested with XbaI and EcoRI and cloned into pUC18NotI-5072Up digested with the same restriction enzymes, resulting in pUC18NotI-5072UpDw. The 570 bp 5072UpDw DNA fragment was digested from pUC18NotI-5072UpDw using NotI and subcloned into the suicide vector pKNG101 a mobilizable suicide vector, hosted routinely in the permissive *E. coli* strain CC118λ*pir* bearing the positive counter selection marker *sacB* and conferring resistance to streptomycin. The resulting plasmid, pKNG101-PA5072UpDw was transformed into *E. coli* CC118λ*pir*. The pKNG101-PA5072UpDw plasmid was mobilized from this strain into *P. aeruginosa* PAO1 by three-partner conjugation using the *E. coli* HB101(pRK600) helper strain. Selection for plasmid cointegration in *P. aeruginosa* PAO1 was accomplished using M9 minimal medium supplemented with 10 mM succinate and 2000 μg/ml streptomycin (Sm). The Sm^r^ colonies were unable to grow on LB medium containing 10% sucrose (Suc), confirming that the plasmid pKNG101-PA5072UpDw with its *sacB* gene had integrated into *P. aeruginosa* PAO1. Transconjugants were plated on LB plates and incubated for 48 h at room temperature. PCR analysis of the Suc^r^ Sm^s^ colonies confirmed that gene replacement had occurred.

### Construction of Plasmid for Complementation

The DNA fragment corresponding to the *mcpK* gene and the 607 bp upstream region was amplified by PCR using primers McpK-comp_fw and McpK-comp_rv and genomic DNA of *P. aeruginosa* PAO1. The resulting fragment was digested with KpnI and XbaI and cloned into pBBR1MCS-2, linearized with the same enzymes. The resulting plasmid pBBR1MCS-2-McpK was verified by DNA sequencing of the insert and flanking regions. The mutant strain was transformed with pBBR1MCS-2-McpK by electroporation (2800 V). Transformed colonies were selected on LB agar plates containing 300 μg/ml kanamycin and verified by colony PCR.

### Quantitative Capillarity Chemotaxis Assays

Assays were carried out at a temperature of 25°C. Bacterial cultures were grown to an OD_600_ of 0.35–0.4 in MS medium, washed and resuspended in chemotaxis buffer (30 mM K_2_HPO_4_, 19 mM KH_2_PO_4_, 20 μM EDTA and 0.05% (v/v) glycerol, pH 7.0) to an OD_600_ of 0.08-0.1. Polystyrene multi-well plates were filled with 230 μl of the resulting bacterial suspension. For filling with chemoeffector solutions, capillaries (Microcaps, Drummond Scientific, USA) were heat-sealed at one end, warmed over the flame and the open end inserted into the chemoattractant solution. The capillary was immersed into the cell suspension at its open end. After incubation for 30 min, the capillary was removed from the cell suspension, rinsed with water and emptied into an Eppendorf tube containing 1 ml M9 medium. Serial dilutions were made and 20 μl aliquots of the resulting cell suspension were plated onto agar plates containing M9 minimal medium supplemented with 15 mM succinate and incubated at 30°C. Colonies were counted after growth for 24 h. Positive (casamino acids) and negative (buffer only) controls were included in each experiment. Data shown are means from three independent experiments conducted in triplicate.

### RNA Extraction and RT-qPCR Analysis

Two flasks with 20 ml of minimal medium M9 containing 10 mM glucose were inoculated with an overnight culture to an OD_600_ of 0.05. At mid-exponential phase, 1 mM of αKG was added to one of the flasks and 0.5 ml samples were taken after 0, 15, 30, and 45 min. Total RNA was extracted using the High Pure RNA Isolation Kit (Roche Diagnostics) according to the manufacturer’s instructions. RNA was treated with Turbo DNAse (Ambion) and the RNA quality and quantity was analyzed by NanoDrop Spectophotometer and agarose gel electrophoresis. cDNA was synthesized from 500 ng of RNA using the SuperScript II Reverse Transcriptase (Invitrogene) and 200 ng of random primers following manufacturers’ instructions. Quantitative PCR was performed using the iQ SYBR green supermix (BIO-RAD) in a MyiQ^TM^2 thermocycler (BIO-RAD). The PCR protocol used was 95°C for 5 min followed by 35 cycles of 95°C (10 s) and 60°C (30 s) and melting curve analysis from 55 to 95°C, with increment of 0.5°C/10 s. Gene expression data were normalized to the expression of the reference gene *rpoD* and reported as normalized fold expression. The primers for *mcpK* and *rpoD* were designed using the Primer3 Plus software.

### Construction of the Strain PAO1-Km

The kamanycin-resistant strain *P. aeruginosa* PAO1-Km was constructed by homologous recombination using a derivative plasmid of the suicide vector pKNG101. The initial plasmid pMAMV257 was generated by separately amplifying the 5′ ends of the convergently transcribed genes *pa5548* and *pa5549* (*glmS*) of *P. aeruginosa* PAO1. These PCR products were obtained using primers glmS-EcoRI_fw and gmlS-BamHI_rv (for amplifying 5′ end of *glmS*) and glmS-BamHI_fw and glmS-HindIII_rv (for amplifying 5′ end of *pa5548*). Subsequently, the PCR products were digested with EcoRI and BamHI (5′ end of *glmS*) or BamHI and HindIII (5′ end of *pa5548*) and ligated in a three-way ligation into pUC18Not. For the generation of the final PAO1-Km strain, the suicide plasmid pMAMV261 was transferred to *P. aeruginosa* PAO1 by triparental conjugation using *E. coli* CC118λ*pir* and *E. coli* HB101 (pRK600) as helper. PAO1 cells, in which pMAMV261 has integrated into the chromosome, were selected on minimal medium containing 400 μg/ml streptomycin and 200 μg/ml kanamycin. To select derivatives that had undergone a second crossover event, sucrose was added to a final concentration of 10% (w/v). The final PAO1-Km strain was confirmed by PCR and sequencing.

### Competitive Root Colonization Assays

Sterilization, germination and inoculation of maize seeds was carried out as described previously, with minor modifications (Matilla et al., 2007). Briefly, sterile seeds were incubated for 1 h at 30°C with a 10^7^ CFU/ml 1:1 mixture of PAO1-Km and Δ*mcpK*. Thereafter, seeds were rinsed with sterile deionized water and planted in 50 ml Sterilin tubes containing 40 g of sterile washed silica sand and 10% (v/w) plant nutrient solution supplemented with Fe-EDTA and micronutrients. Plants were maintained at 24°C with a daily light period of 16 h. After 7 days, bacterial cells were recovered from the rhizosphere or from 1 mm of the main root apex, as described previously (Matilla et al., 2007). Serial dilutions were plated in LB-agar and LB-agar medium supplemented with 400 μg/ml of kanamycin, to select the wild type strain PAO1-Km.

## Results

### α-Ketoglutarate Causes the Most Pronounced Increase in Thermal Stability of the PA5072-LBD

To identify the putative LBD of chemoreceptor PA5072, its sequence was analyzed using the DAS transmembrane prediction algorithm (Cserzo et al., 1997). Two transmembrane regions were identified that flank the segment of amino acids 38–293, which likely corresponds to the periplasmic LBD (**Supplementary Figure S2**). The DNA fragment encoding the PA5072-LBD was cloned into an expression vector, the protein overexpressed in *E. coli* and purified from the soluble fraction of the *E. coli* lysate.

To identify whether and which ligand bind may bind to this domain, we carried out thermal shift assays in high throughput screening format as reported by McKellar et al. (2015). In this assay, a temperature gradient is applied to a mixture of the purified protein and a fluorescent compound. During protein unfolding buried hydrophobic parts of the protein will be exposed leading to additional dye binding, causing fluorescent changes, which is the signal recorded. Consequently, this assay permits the calculation of the Tm value, which corresponds to the temperature at which half of the protein is in its native form and half is in the unfolded form (Krell, 2015). Ligand binding to a protein causes typically Tm changes, which gives useful initial information as to the identification of potential ligands. This assay has been essential to gain initial insight into the specificity of several chemoreceptors (McKellar et al., 2015; Corral-Lugo et al., 2016; Fernandez et al., 2016).

**FIGURE 1:**
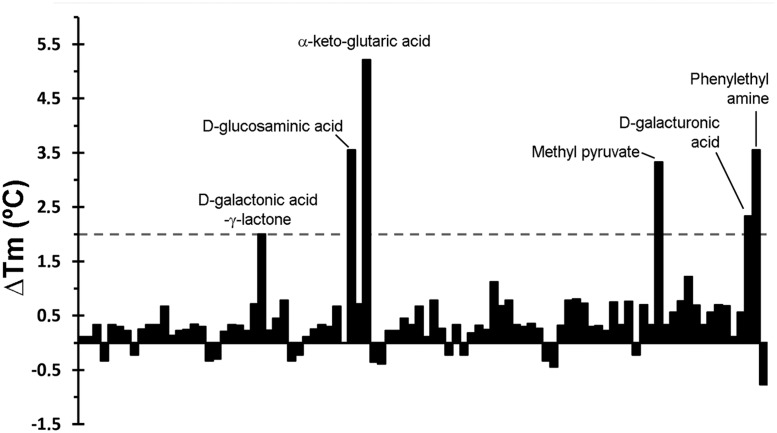
**Thermal shift assays of the recombinant ligand binding domain of the PA5072/McpK chemoreceptor in the presence of bacterial carbon sources from the Biolog screen plate PM1.** Shown are changes in Tm respective to the protein without ligand (38.7°C). A list of the individual compounds in this screen is provided in **Supplementary Figure S1**. Compounds that caused Tm shifts of at least 2°C are annotated.

The thermal shift assays of ligand-free PA5072-LBD resulted in a Tm of 38.7°C. Ligands tested included different bacterial carbon-, nitrogen-, phosphorous-, and sulfur sources as well as to nutrient supplements, which are listed in **Supplementary Figure S1**. As a representative example, the Tm changes induced by compounds of plate PM1 are shown in **Figure 1**. A total of 13 compounds were identified that increased or decreased the Tm by at least 2°C (**Supplementary Table S2**), which is the generally accepted threshold for a relevant ligand-induced change in protein stability. This analysis showed also that α-keto-glutaric acid caused with 5.2°C the most pronounced Tm increase.

### α-Ketoglutarate Binds With Positive Cooperativity to PA5072-LBD

**FIGURE 2:**
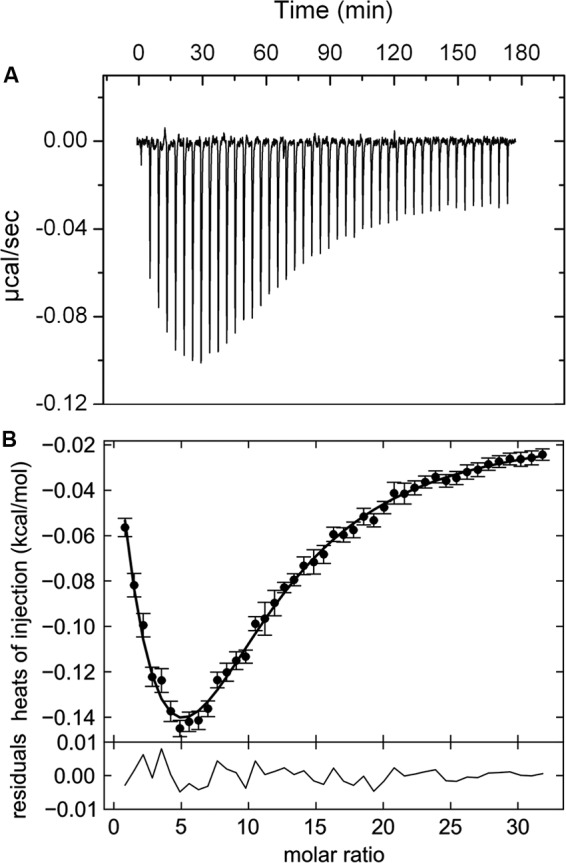
**Isothermal titration calorimetry data for the binding of αKG to the ligand binding domain of the McpK chemoreceptor.** **(A)** Heat changes caused by the injection of 3 mM αKG into 20 μM McpK-LBD. **(B)** Dilution heat-corrected and concentration-normalized integrated peak areas of raw data. The solid line shows the best fit with “the two symmetric-site binding model” of the SEDPHAT program. The residual of the curve fit are shown in the lower part of the figure.

Thermal shift assays provide initial information on ligands that bind but represent no evidence of binding. A valid criterion to ascertain binding are isothermal titration calorimetry (ITC) (Krell, 2008) binding experiments. PA5072-LBD was titrated with all compounds that caused Tm changes of at least 2°C (**Supplementary Table S2**). In these experiments α-ketoglutarate (αKG) was the only compound that showed binding and the corresponding titration data are shown in **Figure 2**. The titration caused exothermic heat changes (down going peaks), but the biphasic titration curve indicates that the reaction is more complex than the binding of a ligand to a single site at a macromolecule. Data analysis was carried out with several models for the dependent or independent binding of ligands to multiple sites. A very satisfactory curve fit was obtained using a model for the cooperative binding of a molecule to two sites. The initial binding event was characterized by a *K*_d1_ of 301.4 ± 0.2 μM (Δ*H*1 = -0.16 ± 0.05 kcal/mol), whereas the second event had a *K*_d2_ of 80.90 ± 0.05 μM (Δ*H*_2_ = -2.99 ± 0.15 kcal/mol). The cooperativity factor μ was of 4.41 (with μ of 1 for a non-cooperative process), indicating an approximately fourfold increase in binding affinity of the second binding event as compared to the initial event. Alpha-KG binds thus with positive cooperativity to PA5072-LBD.

The criterion to establish that a given compound does not bind is the absence of binding heats in experiments conducted at two different analysis temperatures to exclude the possibility that exothermic and endothermic contributions to binding cancel out each other at a given analysis temperature. Therefore, binding of compounds that failed to bind at 20°C were also analyzed at 10°C, which in all cases confirmed the absence of binding. As additional control experiment, αKG was titrated into mixtures of protein with ligands that did not cause binding heats to verify protein integrity. In summary, of the compounds that caused significant Tm shifts, only αKG was confirmed as PA5072 ligand.

We then explored by ITC whether other compounds that are structurally similar to αKG may bind to PA5072-LBD and the 15 ligands selected for further ITC studies are provided in **Supplementary Table S2**. In analogy to the above results, we were unable to detect any binding and concluded that PA5072 binds exclusively αKG. The receptor was therefore named McpK (methyl-accepting chemotaxis protein K).

### McpK-LBD Dimer Stabilization by αKG Binding

**FIGURE 3:**
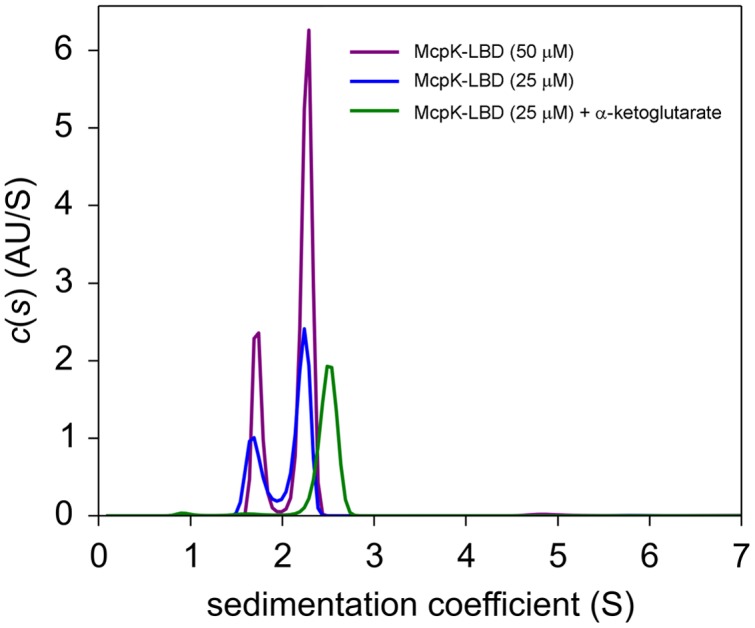
**Determination of the oligomeric state of McpK-LBD by sedimentation velocity analytical ultracentrifugation.** Shown are sedimentation coefficient distributions of McpK-LBD in the absence and presence of 1 mM α-ketoglutarate.

The unexpected observation of binding with positive cooperativity indicates the presence of higher oligomeric states of the protein analyzed. To assess this issue, we carried out analytical ultracentrifugation (AUC) studies of McpK-LBD in the absence and presence of αKG. Initial SM studies of ligand free protein revealed two species with standard sedimentation coefficients of *s_20,w_* = 2.45 S and *s_20,w_* = 3.18 S (**Figure 3**; note: these values are standard values normalized for migration in water, whereas **Figure 3** shows the experimental data recorded in buffer). The frictional ratio (*fr*) for the peak at 2.45 S is 1.5, indicative of a rather elongated protein morphology, which agrees with the structure of HBM domains (Pineda-Molina et al., 2012; Ortega and Krell, 2014). Based on this frictional ratio and using the diffusional scaling law of SEDFIT and the Svedberg equation, the average molar mass of the protein was determined 31.1 kDa, which is very close to the sequence-derived mass of the monomer (30.5 kDa). The peak of the fast sedimenting species (*s_20,w_* = 3.18 S) shows a similar *fr* corresponding to an elongated particle. The molar mass extracted was 45.8 kDa, which may point to a virtual intermediate species resulting from the fast equilibrium between the monomers and dimers. Such virtual species have been observed previously for the analysis of the homologous domain of the *P. putida* KT2440 McpQ chemoreceptor (Martin-Mora et al., 2016). When the experiment was conducted in the presence of 1 mM αKG a single peak with *s_20,w_* = 3.58 S was observed that translates to a species with a molecular mass of 58.7 kDa, close to the sequence derived mass of the protein dimer (61 kDa).

**FIGURE 4:**
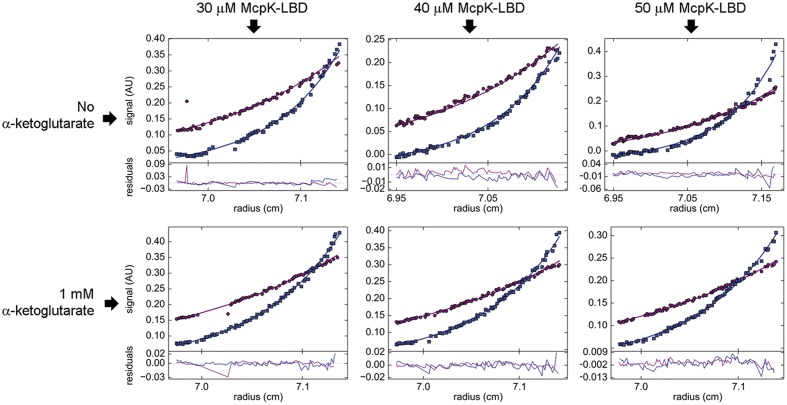
**Study of McpK-LBD dimer association by sedimentation equilibrium analytical ultracentrifugation.** Sedimentation equilibrium profiles of McpK-LBD at a speed of 9,740 (purple) and 20,606 × *g* (blue), at three different protein concentrations and in the absence and presence of 1 mM αKG. The best global fit is shown as a continuous line for both speeds along with the residuals of fitting in the lower part of the figure.

The self-association behavior observed by SM was further demonstrated by multi-speed SE experiments. The SE of McpK at three different concentrations was analyzed by a global fit that confirmed the presence of both the monomeric and dimeric species at all concentrations (**Figure 4**). For ligand-free McpK-LBD a dimer self-dissociation constant of 55.0 μM could be determined. When this experiment was repeated in the presence of 1 mM αKG, a tighter association was observed, with an approximately 10-fold lower self-dissociation constant of 5.9 μM, confirming that αKG causes McpK-LBD dimer stabilization.

At the protein concentration used for ITC binding studies, McpK-LBD is thus partially present as dimer and the cooperativity observed is thus likely due to ligand binding to the different monomers of the dimer in a way that the initial binding to one monomer of the dimer enhances the affinity for the second monomer.

### McpK Mediates Chemotaxis to αKG

Chemoreceptors can either mediate chemotaxis, have alternative cellular functions or are responsible for type IV pili mediated motility (Wuichet and Zhulin, 2010). To determine McpK function, we generated a *mcpK* deletion mutant and carried out quantitative capillary chemotaxis assays. Initially, control experiments were conducted to assess chemotaxis of the wt and mutant strain toward casamino acids. The choice of this chemoattractant was based on the fact that the chemotaxis toward amino acids is mediated by the three well-characterized chemoreceptors PctA, PctB and PctC (Taguchi et al., 1997; Rico-Jimenez et al., 2013). As shown in **Supplementary Figure S3**, deletion of *mcpK* did not alter significantly chemotaxis to casamino acids, indicating that this mutation did not cause any general motility defect.

**FIGURE 5:**
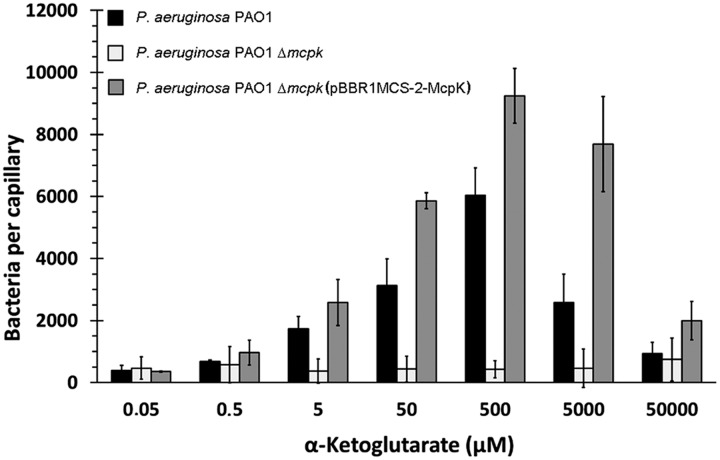
**The effect of McpK on α-ketoglutarate chemotaxis.** Quantitative capillary chemotaxis assays of *P. aeruginosa* PAO1, *P. aeruginosa ΔmcpK* and the complemented mutant *P. aeruginosa*Δ*mcpK* (pBBR1MCS-2-McpK) toward different αKG concentrations. Data were corrected with the number of cells that swam into buffer-containing capillaries (833 ± 106). Shown are means and standard errors from three independent experiments, each conducted in triplicate.

Subsequently, chemotaxis assays toward different αKG concentrations were performed. Experiments showed that the wt strain has significant taxis to αKG concentrations between 5 μM and 5 mM, with an optimal response at 500 μM (**Figure 5**). Importantly, the deletion of the *mcpK* gene resulted in a drop in the chemotactic response to close to baseline levels. Complementation of this mutant by the *in trans* expression of the *mcpK* gene restored wild type like chemotaxis at all concentrations (**Figure 5**). In order to determine whether the observed chemotaxis defect in the *mcpK* mutant is the result of an altered metabolism, we performed growth curves in M9 minimal medium supplemented with αKG and succinate as carbon sources. As shown in **Supplementary Figure S4**, there were no differences between the wt and mutant strain. Taken together, these data show that McpK is the primary chemoreceptor for αKG in *P. aeruginosa* PAO1.

### α-Ketoglutarate Does Not Regulate *mcpK* Expression

We have recently assessed the effect of chemoeffectors on the gene expression of their cognate chemoreceptors in *P. putida* KT2440 (López-Farfán et al., 2016). We were able to show that the expression of a significant number of chemoreceptor genes is either up- or downregulated by the cognate chemoeffectors. However, this was not the case for genes encoding chemoreceptors that respond to TCA cycle intermediates, namely *mcpS*, *mcpQ* and *mcpR* that were expressed independently of the presence or absence of their cognate ligands.

**FIGURE 6:**
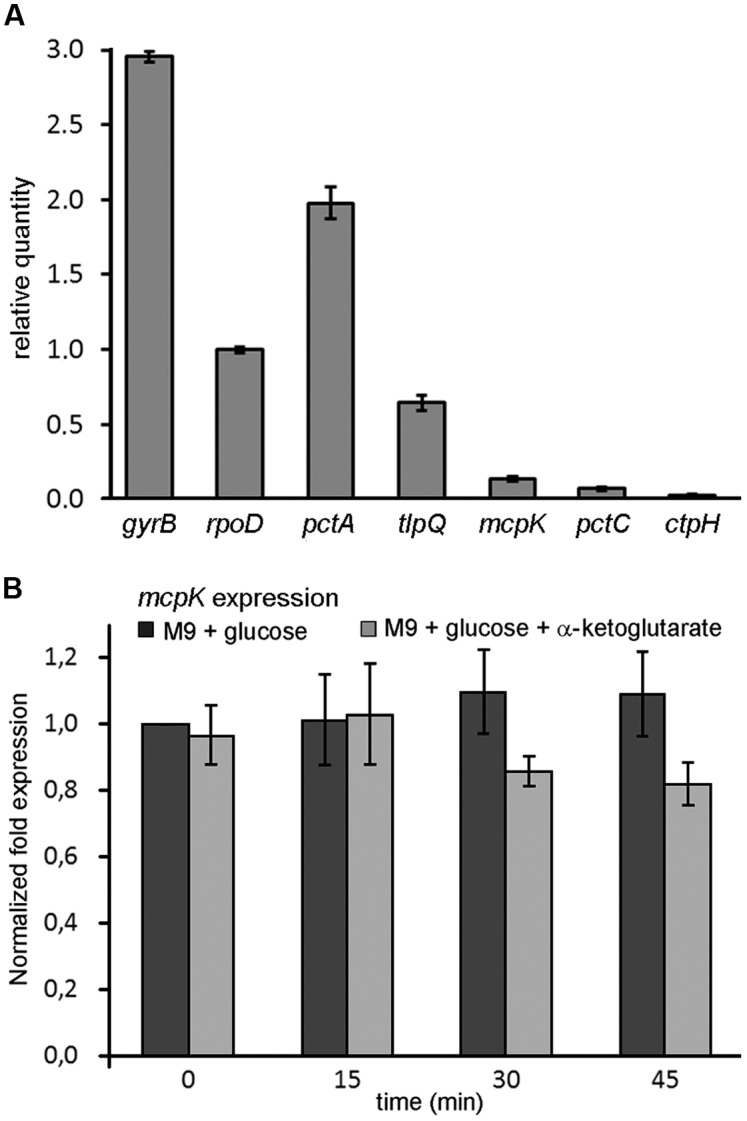
**Expression of the *mcpK* gene in the absence and presence of α-ketoglutarate.** **(A)** RT-qPCR measurements of the relative transcript levels of *P. aeruginosa mcpK, pctA, tlpQ, pctC* and *ctpH* chemoreceptor genes as well as of the housekeeping genes *gyrB* and *rpoD.* Cells were grown in M9 minimal medium supplemented with 10 mM glucose and samples taken at mid-exponential phase. **(B)** Gene expression of *mcpK* in the absence and presence of 1 mM αKG. Cells were grown to mid-exponential phase in M9 supplemented with 10 mM glucose and samples were taken after 0, 15, 30, and 45 min. The results are expressed as the relative expression of *mcpK* normalized with the transcript level of the reference gene *rpoD* at time 0 in the absence of αKG. Data shown are the average of two independent experiments.

To assess *mcpK* expression, we carried out real-time quantitative PCR measurements. **Figure 6A** shows *mcpK* transcript levels of samples taken during mid-exponential phase in comparison to those of the housekeeping genes *gyrB* and *rpoD* as well as to genes encoding functionally characterized chemoreceptors such as the amino acid sensors PctA and PctC (Taguchi et al., 1997), the Pi responsive CtpH (Wu et al., 2000) or the TlpQ chemoreceptor that mediates taxis to ethylene (Kim et al., 2007). Data show that *mcpK* transcript levels are low as compared to the housekeeping genes and in between most and less abundant chemoreceptor transcripts.

In subsequent experiments we assessed the effect of αKG on *mcpK* expression. However, the addition of αKG to *P. aeruginosa* cultures grown in M9 minimal medium supplemented with glucose did not alter *mcpK* expression (**Figure 6B**). Data thus show that, in analogy to the TCA cycle intermediate responsive chemoreceptors in *P. putida* KT2440, the cognate chemoeffector αKG does not modulate *mcpK* expression. Further experiments will show whether the constitutive expression of TCA cycle intermediate responsive chemoreceptors, as observed in *P. putida* and *P. aeruginosa*, is a general feature.

### McpK Mediated Chemotaxis Does Not Affect Plant Root Colonization

*Pseudomonas aeruginosa* is a universal pathogen that is also able to colonize and infect different plants (Rahme et al., 2000; Cao et al., 2001; Walker et al., 2004; Attila et al., 2008). The web-based resource PIFAR allowed the identification of 175 gene products in *P. aeruginosa* putatively involved in the interaction with plants (Martinez-Garcia et al., 2016) and the TlpQ chemoreceptor was found to mediate chemotaxis toward the plant hormone ethylene (Kim et al., 2007). αKG is present at significant levels in plant root exudates (Tawaraya et al., 2014; Ganie et al., 2015) and chemotaxis to root exudate components was shown to be essential for efficient root colonization (de Weert et al., 2002; Scharf et al., 2016).

**FIGURE 7:**
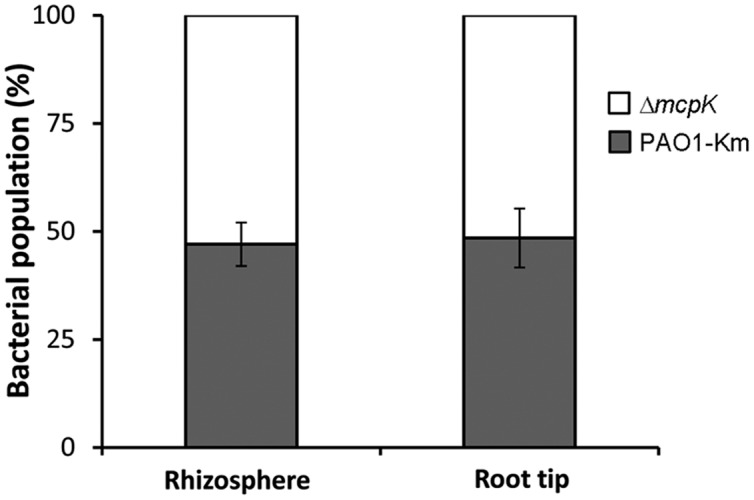
**Competitive root colonization of *Pseudomonas aeruginosa* PAO1-Km and *P. aeruginosa* Δ*mcpK*.** The figure represents the percentage of bacteria recovered either from the rhizosphere or root tips of maize (*Zea mays*) plants. Data are the means and standard deviations of six plants.

To determine the role of McpK in the colonization of the rhizosphere, we performed competitive colonization assays using maize as model plant. Initial experiments showed that *P. aeruginosa* colonizes the maize rhizosphere at a density of around 5 × 10^7^ bacteria per gram of root. To distinguish between the wild type and the mutant strain, a kanamycin-resistant *P. aeruginosa* wild type strain was generated. This strain contains a kanamycin cassette inserted downstream of the glucosamine-6-phosphate synthetase encoding gene, *glmS*. This region was demonstrated to be neutral in multiple *Pseudomonas* strains, including *P. aeruginosa* (Koch et al., 2001; Matilla et al., 2007). The resulting strain, *P. aeruginosa* PAO1-Km, was shown to mediate chemotaxis to αKG (and other known chemoattractants) at the same levels as PAO1 (**Supplementary Figure S5**). Competitive colonization assays showed that the fitness of the mutant in *mcpK* in the rhizosphere was similar to the strain PAO1-Km (**Figure 7**). Additionally, the strain Δ*mcpK* also colonized root tips at the wild type levels (**Figure 7**).

## Discussion

**FIGURE 8:**
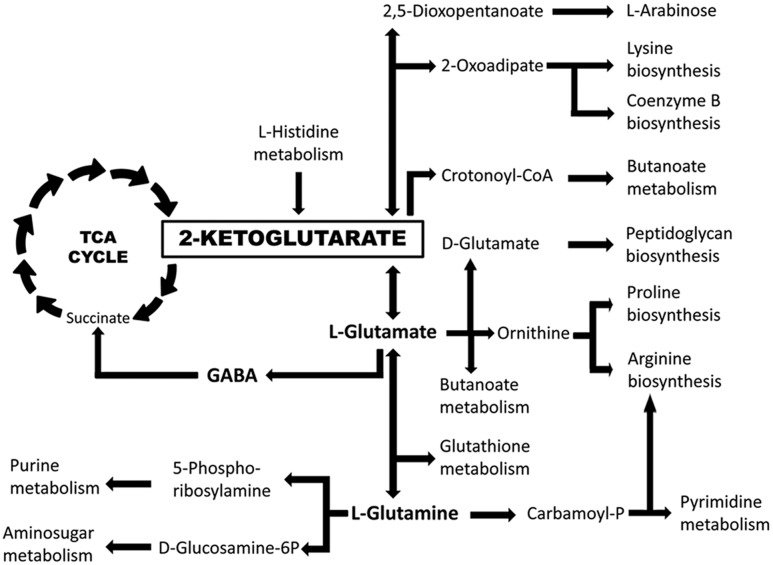
**The central role of αKG in the metabolism of *P. aeruginosa* PAO1.** Implication of αKG in key metabolic pathways as derived from the KEGG (Kanehisa et al., 2016) map of *P. aeruginosa* PAO1.

Chemoreceptors can be classified according to their ligand spectrum into receptors that recognize various, structurally related chemoeffectors and those that appear to be specific for a single chemoeffector. Examples of the former group are receptors for different L-amino acids (Taguchi et al., 1997; Glekas et al., 2012; Oku et al., 2012; Brennan et al., 2013; Rico-Jimenez et al., 2013; Webb et al., 2016), cyclic organic acids (Luu et al., 2015), purines (Fernandez et al., 2016), polyamines (Corral-Lugo et al., 2016), aromatic hydrocarbons (Lacal et al., 2011b), C4–C6 organic acids (Lacal et al., 2010a; Parales et al., 2013) or C2- and C3-organic acids (Garcia et al., 2015). Chemoreceptors that appear to respond to a single compound include the citrate specific chemoreceptors Tcp of *Salmonella typhimurium* (Yamamoto and Imae, 1993) and McpQ of *P. putida* KT2440 (Martin-Mora et al., 2016), the malate specific receptor PA2652 in *P. aeruginosa* or the GABA specific McpG of *P. putida* KT2440 (Reyes-Darias et al., 2015a). Here we report with McpK another chemoreceptor that binds specifically a single compound, αKG. Interestingly, the ligands of these specific chemoreceptors are either part of or closely linked to the TCA cycle (note: GABA is part of the GABA shunt converting αKG into succinate). The evolution of specific chemoreceptors suggests that these compounds are of importance to the microorganism. The existence of a chemoreceptor dedicated to αKG may be linked to the central metabolic role of this compound (**Figure 8**). Apart from being part of the TCA cycle, αKG also represents a branch point from which other important metabolic pathways depart such as the GABA shunt, biosynthetic pathways for several amino acids and coenzyme B or the purine and pyrimidine synthesis.

Several important chemoattractants have a dual function and exert metabolic as well as signaling roles. Examples are GABA, the only ligand of *P. putida* KT2440 McpG (Reyes-Darias et al., 2015a), and putrescine, the high-affinity ligand of McpU of the same species (Corral-Lugo et al., 2016). Both compounds serve as carbon and nitrogen sources and also exert functions as signaling molecules. Alpha-KG belongs to the same class of compound. Apart from being a carbon source it was shown to modulate the activity of the NtrB/NtrC two component system (TCS) for the control of nitrogen utilization processes (Li and Lu, 2007). The activity of this TCS is regulated by the small protein PII that senses αKG as carbon signal and glutamine as nitrogen signals (Ninfa and Jiang, 2005). In addition, a model was proposed in which αKG regulates in *P. aeruginosa* the activity of another TCS, namely the MifS/MifR system, which regulates genes that are involved in αKG transport and subsequent metabolism (Tatke et al., 2015).

In contrast to the dCACHE domain that recognizes ligands in the monomeric state (Rico-Jimenez et al., 2013), the HBM and 4-helix bundle domain need to be dimeric for ligand recognition (Milburn et al., 1991; Lacal et al., 2010a; Martin-Mora et al., 2016). This is due to the fact that the ligand binding sites are at the dimer interface and that amino acids from both monomers of the dimer are involved in ligand binding (Milburn et al., 1991; Pineda-Molina et al., 2012). Tsr (Lin et al., 1994) and Tar (Milligan and Koshland, 1993; Biemann and Koshland, 1994; Danielson et al., 1994) that both contain a 4-helix bundle domain, bind serine and aspartate, respectively, with a 1 per dimer stoichiometry, which is due to an extreme form of negative cooperativity in which ligand binding to the first monomer causes a dramatic reduction of affinity for the second monomer of the dimer. This negative cooperativity has been observed for the full-length receptor (Biemann and Koshland, 1994; Lin et al., 1994) as well as for the individual, recombinant LBDs (Milligan and Koshland, 1993; Danielson et al., 1994). In marked contrast, McpK-LBD bound its ligand with positive cooperativity. In contrast to the very strong negative cooperativity observed for Tar and Tsr, the positive cooperativity at McpK-LBD was more modest and ligand binding at the first monomer increased affinity of the second ligand by approximately fourfold. To our knowledge, this is the first report on a chemoreceptor-LBD that recognizes its ligands in positively cooperative manner. The evolution of a receptor with positive cooperativity may be straightforward, since it was shown that the mutation of a single amino acid at the dimer interface of Tar converts its negative cooperativity into positive cooperativity (Kolodziej et al., 1996). Further experiments will provide insight as to the functional or physiological reasons for ligand recognition with positive cooperativity.

Analytical ultracentrifugation studies show that αKG binding stabilizes the dimer. Equilibrium studies showed that αKG binding reduces the dimer self-dissociation constant from 55 to 5.9 μM. Chemoeffector mediated LBD dimer stabilization appears to be a general feature of 4-helix bundle and HBM domains since similar observations have been made for Tar-LBD (Milligan and Koshland, 1993; Yu et al., 2015), McpQ (Martin-Mora et al., 2016), McpS (Lacal et al., 2010a).

The expression of the genes of a significant number of *P. putida* chemoreceptors is modulated by their cognate ligands (López-Farfán et al., 2016). However, exceptions were the TCA cycle intermediate responsive chemoreceptor genes that are expressed constitutively. It was hypothesized that the omnipresence in natural habitats and metabolic importance of TCA cycle intermediates may be responsible for this constitutive expression (López-Farfán et al., 2016). The present finding that αKG does not modulate *mcpK* expression provides further support to this hypothesis.

Chemotaxis to plant root exudates was shown to promote bacterial colonization (de Weert et al., 2002; Reyes-Darias et al., 2015a; Scharf et al., 2016). However, the deletion of the *mcpK* gene did not cause any significant differences in maize root colonization. Root exudates are complex mixtures of mainly sugars, amino acids and organic acids. A significant part of *P. aeruginosa* chemoreceptors are likely to respond to exudate components (like PctA, PctB and PctC) and therefore the effects caused by the elimination of a single chemoreceptor is compensated by other root exudate responsive receptors. We have shown that the elimination of the GABA specific McpG receptor did reduce root colonization (Reyes-Darias et al., 2015a). However, GABA recognition at McpG-LBD (*K*_D_ = 175 nM) was much tighter than αKG binding to McpK-LBD (*K*_d1_ = 301 μM, *K*_d2_ = 81 μM).

Chemoreceptors contain a variety of different LBD types (Lacal et al., 2010b; Upadhyay et al., 2016) and a central question in understanding this diversity is to elucidate whether there is a relationship between the LBD type and the structure of the chemoeffector recognized. In this aspect first tendencies have appeared and it was suggested that sCACHE domains may be linked to the recognition of C2- and C3-carboxylic acids (Garcia et al., 2015), whereas the dCACHE domain may be the dominant domain for the recognition of L-amino acids (Glekas et al., 2012; Oku et al., 2012; Liu et al., 2015; Reyes-Darias et al., 2015b). The alignment of all members of the HBM domain family revealed the conservation of amino acids of the ligand binding cavity (Ortega and Krell, 2014). Since C4 to C6 organic acids were found to bind to the first characterized member of this family, McpS, it was proposed that the HBM domain may be associated with the recognition of organic acids (Ortega and Krell, 2014). This hypothesis was supported by the identification of other HBM domain containing chemoreceptors that mediate taxis to this class of compound such as the citrate specific McpQ as well as McfS/McfQ (Parales et al., 2013) and Pfl01_0728 that all respond to organic acids (Oku et al., 2014). The identification of McpK as another HBM family member that binds to an organic acid lends further support to this hypothesis. Establishing LBD type – chemoeffector relationships will permit to orient experiments to a certain group of compounds, which in turn will accelerate the functional annotation of receptors.

## Author Contributions

DM-M, AO, JR-D, VG, and DL-F designed experiments, conducted experiments and analyzed data; MM conducted research, analyzed data and wrote the manuscript; TK designed experiments, analyzed data and wrote the manuscript.

## Conflict of Interest Statement

The authors declare that the research was conducted in the absence of any commercial or financial relationships that could be construed as a potential conflict of interest. The reviewer BES and handling Editor declared their shared affiliation, and the handling Editor states that the process nevertheless met the standards of a fair and objective review.
