# Supplementary material for: Identification of a Chemoreceptor in Pseudomonas aeruginosa That Specifically Mediates Chemotaxis Toward α-Ketoglutarate
Source: Front Microbiol. 2016 Nov 29;7:1937. doi: 10.3389/fmicb.2016.01937 (PMC5126104; doi:10.3389/fmicb.2016.01937)
Supplement: Supplementary file 1 [file Presentation_1.PDF]

Supplementary Material

to

Identification of a Chemoreceptor in *Pseudomonas aeruginosa* that specifically mediates Chemotaxis towards α-Ketoglutarate

David Martín-Mora, Alvaro Ortega, José Antonio Reyes-Darias, Vanina García, Diana López-Farfán, Miguel A. Matilla and Tino Krell

Fig. S1) Composition of Biolog Screens used for the high-throughput ligand screening of McpK-LBD.

Screen PM 1.

A1 Negative Control	A2 L-Arabinose	A3 N-Acetyl-D-Glucosamine	A4 D-Saccharic Acid	A5 Succinic Acid	A6 D-Galactose	A7 L-Aspartic Acid	A8 L-Proline	A9 D-Alanine	A10 D-Trehalose	A11 D-Mannose	A12 Dulcitol
B1 D-Serine	B2 D-Sorbitol	B3 Glycerol	B4 L-Fucose	B5 D-Glucuronic Acid	B6 D-Gluconic Acid	B7 D,L-α-Glycerol-Phosphate	B8 D-Xylose	B9 L-Lactic Acid	B10 Formic Acid	B11 D-Mannitol	B12 L-Glutamic Acid
C1 D-Glucose-6-Phosphate	C2 D-Galactonic Acid-γ-Lactone	C3 D,L-Malic Acid	C4 D-Ribose	C5 Tween 20	C6 L-Rhamnose	C7 D-Fructose	C8 Acetic Acid	C9 α-D-Glucose	C10 Maltose	C11 D-Melibiose	C12 Thymidine
D-1 L-Asparagine	D2 D-Aspartic Acid	D3 D-Glucosaminic Acid	D4 1,2-Propanediol	D5 Tween 40	D6 α-Keto-Glutaric Acid	D7 α-Keto-Butyric Acid	D8 α-Methyl-D-Galactoside	D9 α-D-Lactose	D10 Lactulose	D11 Sucrose	D12 Uridine
E1 L-Glutamine	E2 m-Tartaric Acid	E3 D-Glucose-1-Phosphate	E4 D-Fructose-6-Phosphate	E5 Tween 80	E6 α-Hydroxy Glutaric Acid-γ-Lactone	E7 α-Hydroxy Butyric Acid	E8 β-Methyl-D-Glucoside	E9 Adonitol	E10 Maltotriose	E11 2-Deoxy Adenosine	E12 Adenosine
F1 Glycyl-L-Aspartic Acid	F2 Citric Acid	F3 m-Inositol	F4 D-Threonine	F5 Fumaric Acid	F6 Bromo Succinic Acid	F7 Propionic Acid	F8 Mucic Acid	F9 Glycolic Acid	F10 Glyoxylic Acid	F11 D-Cellobiose	F12 Inosine
G1 Glycyl-L-Glutamic Acid	G2 Tricarballylic Acid	G3 L-Serine	G4 L-Threonine	G5 L-Alanine	G6 L-Alanyl-Glycine	G7 Acetoacetic Acid	G8 N-Acetyl-β-D-Mannosamine	G9 Mono Methyl Succinate	G10 Methyl Pyruvate	G11 D-Malic Acid	G12 L-Malic Acid
H1 Glycyl-L-Proline	H2 p-Hydroxy Phenyl Acetic Acid	H3 m-Hydroxy Phenyl Acetic Acid	H4 Tyramine	H5 D-Psicose	H6 L-Lyxose	H7 Glucuronamide	H8 Pyruvic Acid	H9 L-Galactonic Acid-γ-Lactone	H10 D-Galacturonic Acid	H11 Phenylethyl-amine	H12 2-Aminoethanol

Screen PM2A.

A1<br>Negative Control	A2<br>Chondroitin Sulfate C	A3<br>α-Cyclodextrin	A4<br>β-Cyclodextrin	A5<br>γ-Cyclodextrin	A6<br>Dextrin	A7<br>Gelatin	A8<br>Glycogen	A9<br>Inulin	A10<br>Laminarin	A11<br>Mannan	A12<br>Pectin
B1<br>N-Acetyl-D-Galactosamine	B2<br>N-Acetyl-Neuraminic Acid	B3<br>β-D-Allose	B4<br>Amygdalin	B5<br>D-Arabinose	B6<br>D-Arabitol	B7<br>L-Arabitol	B8<br>Arbutin	B9<br>2-Deoxy-D-Ribose	B10<br>i-Erythritol	B11<br>D-Fucose	B12<br>3-0-β-D-Galacto-pyranosyl-D-Arabinose
C1<br>Gentiobiose	C2<br>L-Glucose	C3<br>Lactitol	C4<br>D-Melezitose	C5<br>Maltitol	C6<br>a-Methyl-D-Glucoside	C7<br>β-Methyl-D-Galactoside	C8<br>3-Methyl Glucose	C9<br>β-Methyl-D-Glucuronic Acid	C10<br>α-Methyl-D-Mannoside	C11<br>β-Methyl-D-Xyloside	C12<br>Palatinose
D1<br>D-Raffinose	D2<br>Salicin	D3<br>Sedoheptulosan	D4<br>L-Sorbose	D5<br>Stachyose	D6<br>D-Tagatose	D7<br>Turanose	D8<br>Xylitol	D9<br>N-Acetyl-D-Glucosaminitol	D10<br>γ-Amino Butyric Acid	D11<br>δ-Amino Valeric Acid	D12<br>Butyric Acid
E1<br>Capric Acid	E2<br>Caproic Acid	E3<br>Citraconic Acid	E4<br>Citramalic Acid	E5<br>D-Glucosamine	E6<br>2-Hydroxy Benzoic Acid	E7<br>4-Hydroxy Benzoic Acid	E8<br>β-Hydroxy Butyric Acid	E9<br>γ-Hydroxy Butyric Acid	E10<br>a-Keto-Valeric Acid	E11<br>Itaconic Acid	E12<br>5-Keto-D-Gluconic Acid
F1<br>D-Lactic Acid Methyl Ester	F2<br>Malonic Acid	F3<br>Melibionic Acid	F4<br>Oxalic Acid	F5<br>Oxalomalic Acid	F6<br>Quinic Acid	F7<br>D-Ribono-1,4-Lactone	F8<br>Sebacic Acid	F9<br>Sorbic Acid	F10<br>Succinamic Acid	F11<br>D-Tartaric Acid	F12<br>L-Tartaric Acid
G1<br>Acetamide	G2<br>L-Alaninamide	G3<br>N-Acetyl-L-Glutamic Acid	G4<br>L-Arginine	G5<br>Glycine	G6<br>L-Histidine	G7<br>L-Homoserine	G8<br>Hydroxy-L-Proline	G9<br>L-Isoleucine	G10<br>L-Leucine	G11<br>L-Lysine	G12<br>L-Methionine
H1<br>L-Ornithine	H2<br>L-Phenylalanine	H3<br>L-Pyroglutamic Acid	H4<br>L-Valine	H5<br>D,L-Carnitine	H6<br>Sec-Butylamine	H7<br>D.L-Octopamine	H8<br>Putrescine	H9<br>Dihydroxy Acetone	H10<br>2,3-Butanediol	H11<br>2,3-Butanone	H12<br>3-Hydroxy 2-Butanone

Screen PM3B

A1<br>Negative Control	A2<br>Ammonia	A3<br>Nitrite	A4<br>Nitrate	A5<br>Urea	A6<br>Biuret	A7<br>L-Alanine	A8<br>L-Arginine	A9<br>L-Asparagine	A10<br>L-Aspartic Acid	A11<br>L-Cysteine	A12<br>L-Glutamic Acid
B1<br>L-Glutamine	B2<br>Glycine	B3<br>L-Histidine	B4<br>L-Isoleucine	B5<br>L-Leucine	B6<br>L-Lysine	B7<br>L-Methionine	B8<br>L-Phenylalanine	B9<br>L-Proline	B10<br>L-Serine	B11<br>L-Threonine	B12<br>L-Tryptophan
C1<br>L-Tyrosine	C2<br>L-Valine	C3<br>D-Alanine	C4<br>D-Asparagine	C5<br>D-Aspartic Acid	C6<br>D-Glutamic Acid	C7<br>D-Lysine	C8<br>D-Serine	C9<br>D-Valine	C10<br>L-Citrulline	C11<br>L-Homoserine	C12<br>L-Ornithine
D-1<br>N-Acetyl-L-Glutamic Acid	D2<br>N-Phthaloyl-L-Glutamic Acid	D3<br>L-Pyroglutamic Acid	D4<br>Hydroxylamine	D5<br>Methylamine	D6<br>N-Amylamine	D7<br>N-Butylamine	D8<br>Ethylamine	D9<br>Ethanolamine	D10<br>Ethylenediamine	D11<br>Putrescine	D12<br>Agmatine
E1<br>Histamine	E2<br>β-Phenylethyl-amine	E3<br>Tyramine	E4<br>Acetamide	E5<br>Formamide	E6<br>Glucuronamide	E7<br>D,L-Lactamide	E8<br>D-Glucosamine	E9<br>D-Galactosamine	E10<br>D-Mannosamine	E11<br>N-Acetyl-D-Glucosamine	E12<br>N-Acetyl-D-Galactosamine
F1<br>N-Acetyl-D-Mannosamine	F2<br>Adenine	F3<br>Adenosine	F4<br>Cytidine	F5<br>Cytosine	F6<br>Guanine	F7<br>Guanosine	F8<br>Thymine	F9<br>Thymidine	F10<br>Uracil	F11<br>Uridine	F12<br>Inosine
G1<br>Xanthine	G2<br>Xanthosine	G3<br>Uric Acid	G4<br>Alloxan	G5<br>Allantoin	G6<br>Parabanic Acid	G7<br>D,L-α-Amino-N-Butyric Acid	G8<br>γ-Amino-N-Butyric Acid	G9<br>ε-Amino-N-Caproic Acid	G10<br>D,L-α-Amino-Caprylic Acid	G11<br>δ-Amino-N-Valeric Acid	G12<br>α-Amino-N-Valeric Acid
H1<br>Ala-Asp	H2<br>Ala-Gln	H3<br>Ala-Glu	H4<br>Ala-Gly	H5<br>Ala-His	H6<br>Ala-Leu	H7<br>Ala-Thr	H8<br>Gly-Asn	H9<br>Gly-Gln	H10<br>Gly-Glu	H11<br>Gly-Met	H12<br>Met-Ala

Screen PM4A

A1 Negative Control	A2 Phosphate	A3 Pyrophosphate	A4 Trimeta-phosphate	A5 Tripoly-phosphate	A6 Triethyl Phosphate	A7 Hypophosphite	A8 Adenosine- 2'-monophosphate	A9 Adenosine- 3'-monophosphate	A10 Adenosine- 5'-monophosphate	A11 Adenosine- 2',3'-cyclic monophosphate	A12 Adenosine- 3',5'-cyclic monophosphate
B1 Thiophosphate	B2 Dithiophosphate	B3 D,L-α-Glycerol Phosphate	B4 β-Glycerol Phosphate	B5 Carbamyl Phosphate	B6 D-2-Phospho-Glyceric Acid	B7 D-3-Phospho-Glyceric Acid	B8 Guanosine- 2'-monophosphate	B9 Guanosine- 3'-monophosphate	B10 Guanosine- 5'-monophosphate	B11 Guanosine- 2',3'-cyclic monophosphate	B12 Guanosine- 3',5'-cyclic monophosphate
C1 Phosphoenol Pyruvate	C2 Phospho-Glycolic Acid	C3 D-Glucose-1-Phosphate	C4 D-Glucose-6-Phosphate	C5 2-Deoxy-D-Glucose 6-Phosphate	C6 D-Glucosamine-6-Phosphate	C7 6-Phospho-Gluconic Acid	C8 Cytidine- 2'-monophosphate	C9 Cytidine- 3'-monophosphate	C10 Cytidine- 5'-monophosphate	C11 Cytidine- 2',3'-cyclic monophosphate	C12 Cytidine- 3',5'-cyclic monophosphate
D1 D-Mannose-1-Phosphate	D2 D-Mannose-6-Phosphate	D3 Cysteamine-S-Phosphate	D4 Phospho-L-Arginine	D5 O-Phospho-D-Serine	D6 O-Phospho-L-Serine	D7 O-Phospho-L-Threonine	D8 Uridine- 2'-monophosphate	D9 Uridine- 3'-monophosphate	D10 Uridine- 5'-monophosphate	D11 Uridine- 2',3'-cyclic monophosphate	D12 Uridine- 3',5'-cyclic monophosphate
E1 O-Phospho-D-Tyrosine	E2 O-Phospho-L-Tyrosine	E3 Phosphocreatine	E4 Phosphoryl Choline	E5 O-Phosphoryl-Ethanolamine	E6 Phosphono Acetic Acid	E7 2-Aminoethyl Phosphonic Acid	E8 Methylene Diphosphonic Acid	E9 Thymidine- 3'-monophosphate	E10 Thymidine- 5'-monophosphate	E11 Inositol Hexaphosphate	E12 Thymidine 3',5'-cyclic monophosphate
F1 Negative Control	F2 Sulfate	F3 Thiosulfate	F4 Tetrathionate	F5 Thiophosphate	F6 Dithiophosphate	F7 L-Cysteine	F8 D-Cysteine	F9 L-Cysteinyl-Glycine	F10 L-Cysteic Acid	F11 Cysteamine	F12 L-Cysteine Sulfinic Acid
G1 N-Acetyl-L-Cysteine	G2 S-Methyl-L-Cysteine	G3 Cystathionine	G4 Lanthionine	G5 Glutathione	G6 D,L-Ethionine	G7 L-Methionine	G8 D-Methionine	G9 Glycyl-L-Methionine	G10 N-Acetyl-D,L-Methionine	G11 L- Methionine Sulfoxide	G12 L-Methionine Sulfone
H1 L-Djenkolic Acid	H2 Thiourea	H3 1-Thio-β-D-Glucose	H4 D,L-Lipoamide	H5 Taurocholic Acid	H6 Taurine	H7 Hypotaurine	H8 p-Amino Benzene Sulfonic Acid	H9 Butane Sulfonic Acid	H10 2-Hydroxyethane Sulfonic Acid	H11 Methane Sulfonic Acid	H12 Tetramethylene Sulfone

Screen PM5

A1 Negative Control	A2 Positive Control	A3 L-Alanine	A4 L-Arginine	A5 L-Asparagine	A6 L-Aspartic Acid	A7 L-Cysteine	A8 L-Glutamic Acid	A9 Adenosine-3',5'-cyclic monophosphate	A10 Adenine	A11 Adenosine	A12 2'-Deoxy Adenosine
B1 L-Glutamine	B2 Glycine	B3 L-Histidine	B4 L-Isoleucine	B5 L-Leucine	B6 L-Lysine	B7 L-Methionine	B8 L-Phenylalanine	B9 Guanosine-3',5'-cyclic monophosphate	B10 Guanine	B11 Guanosine	B12 2'-Deoxy Guanosine
C1 L-Proline	C2 L-Serine	C3 L-Threonine	C4 L-Tryptophan	C5 L-Tyrosine	C6 L-Valine	C7 L-isoleucine + L-Valine	C8 trans-4-Hydroxy L-Proline	C9 (5) 4-Amino-Imidazole-4(5)-Carboxamide	C10 Hypoxanthine	C11 Inosine	C12 2'-Deoxy Inosine
D1 L-Ornithine	D2 L-Citrulline	D3 Chorismic Acid	D4 (-)Shikimic Acid	D5 L-Homoserine Lactone	D6 D-Alanine	D7 D-Aspartic Acid	D8 D-Glutamic Acid	D9 D,L-α,ε-Diamino-pimelic Acid	D10 Cytosine	D11 Cytidine	D12 2'-Deoxy Cytidine
E1 Putrescine	E2 Spermidine	E3 Spermine	E4 Pyridoxine	E5 Pyridoxal	E6 Pyridoxamine	E7 β-Alanine	E8 D-Pantothenic Acid	E9 Orotic Acid	E10 Uracil	E11 Uridine	E12 2'-Deoxy Uridine
F1 Quinolinic Acid	F2 Nicotinic Acid	F3 Nicotinamide	F4 β-Nicotinamide Adenine Dinucleotide	F5 δ-Amino-Levulinic Acid	F6 Hematin	F7 Deferoxamine Mesylate	F8 D-(+)-Glucose	F9 N-Acetyl D-Glucosamine	F10 Thymine	F11 Glutathione (reduced form)	F12 Thymidine
G1 Oxaloacetic Acid	G2 D-Biotin	G3 Cyano-Cobalamine	G4 p-Amino-Benzoic Acid	G5 Folic Acid	G6 Inosine + Thiamine	G7 Thiamine	G8 Thiamine Pyrophosphate	G9 Riboflavin	G10 Pyrrolo-Quinoline Quinone	G11 Menadione	G12 m-Inositol
H1 Butyric Acid	H2 D,L-α-Hydroxy-Butyric Acid	H3 α-Keto-Butyric Acid	H4 Caprylic Acid	H5 D,L-α-Lipoic Acid (oxidized form)	H6 D,L-Mevalonic Acid	H7 D,L-Carnitine	H8 Choline	H9 Tween 20	H10 Tween 40	H11 Tween 60	H12 Tween 80

A

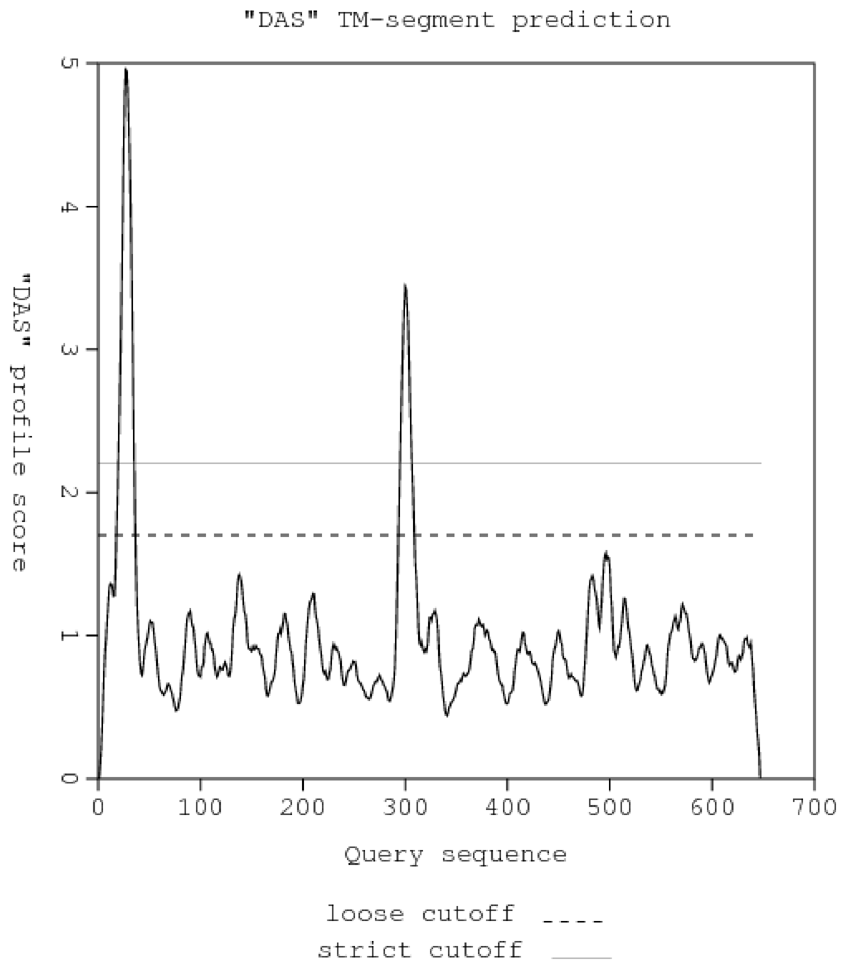

B

```
>PA5072/McpK.
MYDWWVLQLAKLSVSRKLMVGFGVLLALLLLVVISSNRTLTHQTALSEQLAEVASLMEQT
QQAEQGRLAFEAGSDPRQAEQVRQTLAGMLQRLQALRDSELDPAALAHQVEAIEAYRKAF
DDLAAADQQRSAARGVLVGTAQQALDSFARLEELMDASLAQQAGDPQALQRSRAVADLHQ
QLLMVRYQVRGYVFERSDKAEQAAFAAFDALRQAATTLRGQLPGEADAALEQAMGSLQGY
RGGIEQFRAGVIRTRQAQQAMQSSTQDMARAGRTLTEAGRQLRESTASRDRASLWLIAAL
ALAFGCVAGWAINRQIVRPLDEALAQAEAIAAGDLGKRPQNPLTLQRRDELGQLQRVMQR
MGDSLRELVGRISDGVSQLASSAEELSAVTEQTRAGVNSQKVETDQVATAMHEMAATVQD
VARNAELASQAARQADEEARQGDAVVDQAVTRIERLASEMDVSSEAMARLKNESEQIGSV
LDVIKSVAEQTNLLALNAAIEAARAGDAGRGFAVVADEVRGLAQRTQQSTAEIEGLIQRL
QQGAGEAAERLENSRSLTASTVELARRAGAALDSITRTVSDIQNMNLQIATAAEQQSTVA
EEINRSVLSVRDVAEQSAAASEQTAASSGELARLGTQLQAQVGRFRL
```

Fig. S2) Prediction of transmembrane regions of chemoreceptor PA5072 from *P. aeruginosa* PAO1. A) Output from the DAS transmembrane (Cserzo et al. (1997) *Protein Engineering* **10**, 673-676) prediction server. B) Sequence of the PA5072/McpK chemoreceptor with the two transmembrane regions predicted highlighted in red.

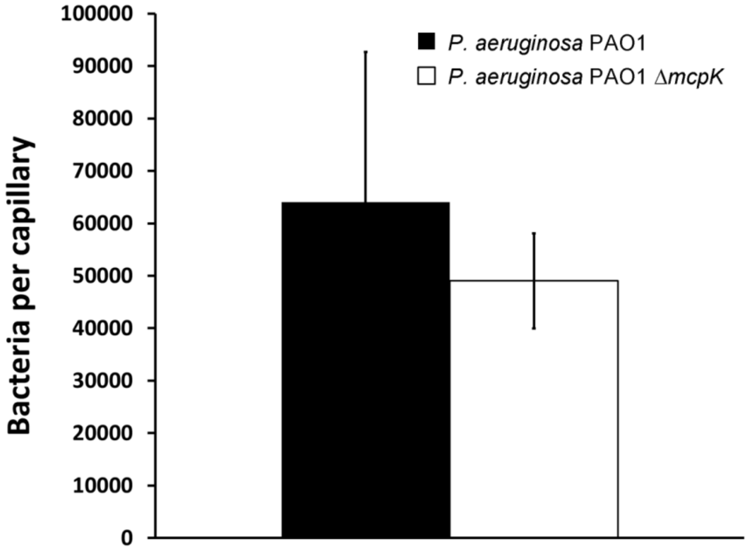

Fig. S3) Quantitative capillary chemotaxis assays of *P. aeruginosa* towards 0.1 % (w/v) casamino acids. Shown are means and standard deviations from three independent experiments conducted in triplicate.

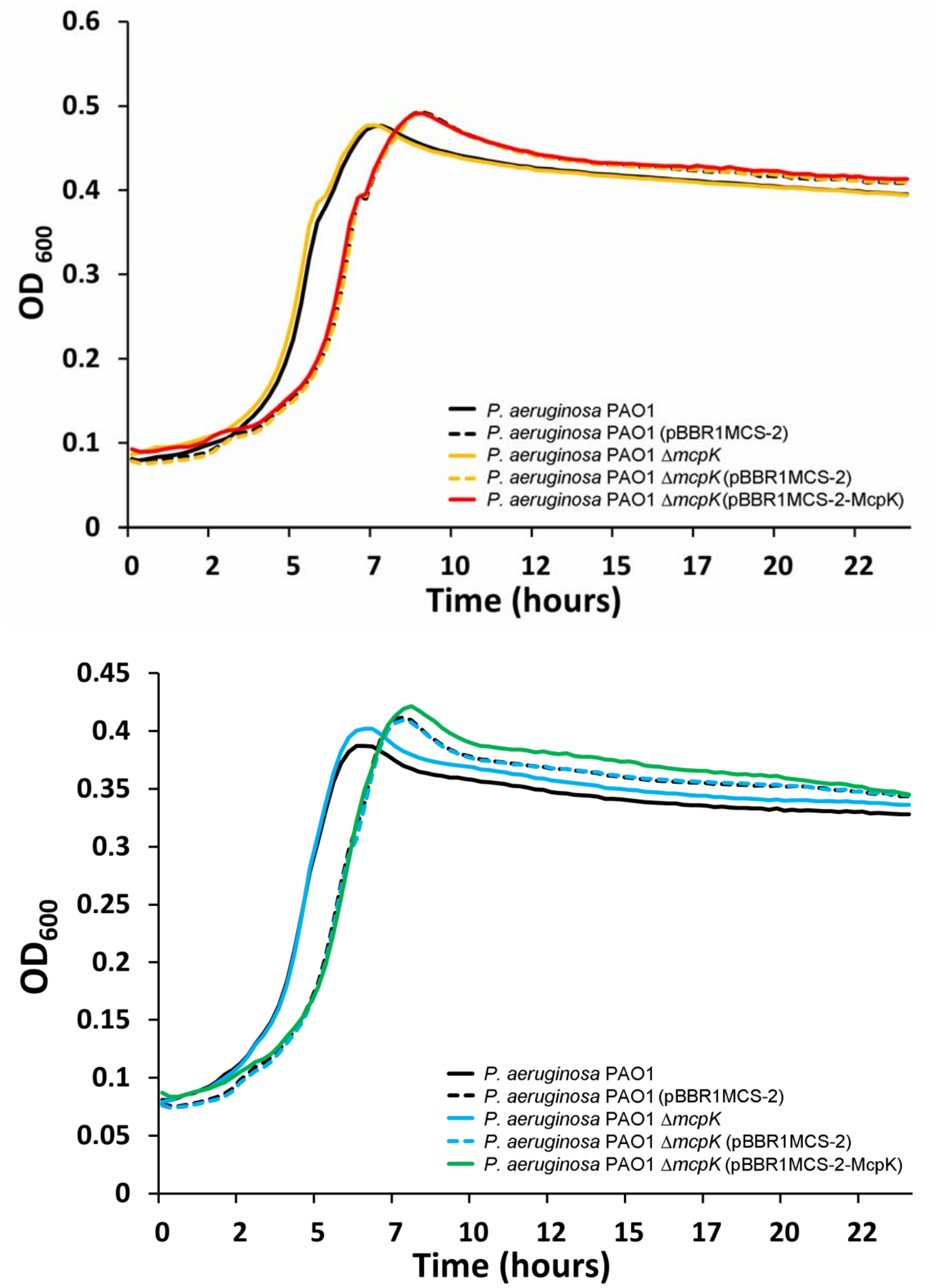

Fig. S4) Growth curve of different strains in M9 minimal medium supplemented with 10 mM α-ketoglutarate (upper graph) or succinate (lower graph).

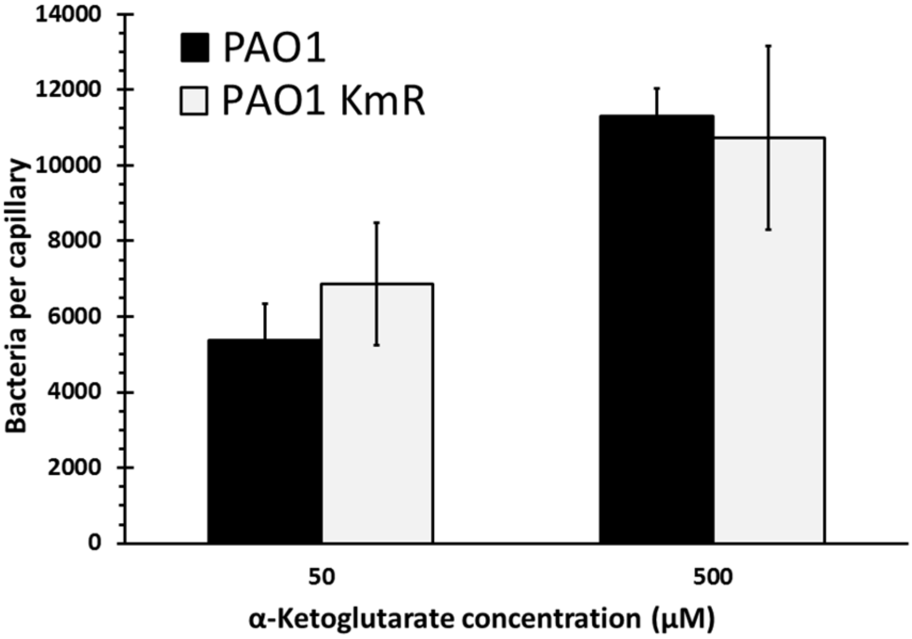

Fig. S5) Chemotaxis of *P. aeruginosa* PAO1 and *P. aeruginosa* PAO1-Km to alpha-ketoglutarate. Shown are means and standard deviations from two experiments conducted in triplicates.

Name	Sequence (5'-3')	Purpose
McpK-LBD_fw	CATATGAGCAACCGCACCCTCACG	Cloning of McpK-LBD into expression vector
McpK-LBD_rv	GGATCCTTAGCTGGCGCGGTCG	Cloning of McpK-LBD into expression vector
PA5072UpF-HindIII	ATAAAGCTTAAAAACTCCGGTAAAAGACTGAAGGT	Generation of Δ*mcpK* mutant
PA5072UpR-XbaI	CTAGTCTAGACTAGCGTCATTACTCCAGGCAGTGCG	Generation of Δ*mcpK* mutant
PA5072DownF-XbaI	CTAGTCTAGACTAGGACATCCAGAACATGAACCTGCA	Generation of Δ*mcpK* mutant
PA5072DownR-EcoRI	AAGAATTCTGGCGTCATCTCCCAAAAGGC	Generation of Δ*mcpK* mutant
glmS-EcoRI_fw	TAATGAATTCGTGCGCGAATCCGACCTGAC	Generation of PAO1-Km strain
glmS-BamHI_rv	TAATGGATCCCGGCGTTACTCGACGGTGAC	Generation of PAO1-Km strain
glmS-BamHI_fw	TAATGGATCCGCTCGCTGTCAATCGCGCAAC	Generation of PAO1-Km strain
glmS-HindIII_rv	TAATAAGCTTGCTGGTCTTCCTGATGGCACG	Generation of PAO1-Km strain
McpK-comp_fw	TAATGGTACCGTGCTGCTGAACAGCTACCG	Generation of complementation plasmid
McpK-comp_rv	TAATTCTAGACCCAAAAGGCGAAAGCCTGA	Generation of complementation plasmid
mcpK_fw	TGCTTCTGCTGGTGGTGATC	qRT-PCR of the *mcpK* gene
mcpK_rv	TTGCTGGGTCTGTTCCATCAG	qRT-PCR of the *mcpK* gene
rpoD_fw	AGAAGAAAGCGACGACAGCA	qRT-PCR of the *rpoD* gene
rpoD_rv	CTTCTTGGCCTTGTCGAGCT	qRT-PCR of the *rpoD* gene
gyrB_fw	CTGAACACCAACAAGACCGC	qRT-PCR of the *gyrB* gene
gyrB_rv	TCGTTGAAGCTGTCGTTCCA	qRT-PCR of the *gyrB* gene
tlpQ_fw	TGAAAAGCGCCAGTACACAG	qRT-PCR of the *tlpQ* gene
tlpQ_rv	CCATGAAATAGCGCTGGATGC	qRT-PCR of the *tlpQ* gene
ctpH_fw	CGAAGACGTGATGGAAGAAACG	qRT-PCR of the *ctpH* gene
ctpH_rv	TTTCCAATTGGCGGATGACC	qRT-PCR of the *ctpH* gene
pctA_fw	TTCGCACTGTTCACCCTCTAC	qRT-PCR of the *pctA* gene
pctA_rv	ATGTTGCTGGAAGTCACGTC	qRT-PCR of the *pctA* gene
pctC_fw	TTTTCGCCTTCAGCTGCTTC	qRT-PCR of the *pctC* gene
pctC_rv	TTTCCCCGAGGTAGTTTTCCG	qRT-PCR of the *pctC* gene

Table S1) Oligonucleotides used in this study

Ligand	Tm Shift (°C)	ITC binding to recombinant McpK-LBD
**Compounds that caused Tm shifts of at least 2 °C**		
α-ketoglutarate	+5.2	Yes
Uracil	+4.2	No
γ-Aminobutyric acid	+4.1	No
5-Aminovaleric acid	+3.9	No
Carbamyl phosphate	+3.5	No
Phenylethylamine	+3.5	No
D-Glucosaminic acid	+3.5	No
Methyl pyruvate	+3.3	No
L-Glutamic acid	+2.3	No
D-Galacturonic acid	+2.3	No
L-Glutamine	+2.2	No
D-Galactonic acid-γ-lactone	+2	No
Itaconate	-2.2	No
**Structurally related compounds**		
Citrate	+0.8	No
Butyrate	+0.7	No
Malate	+0.5	No
Fumarate	+0.3	No
Tricarballylate	+0.3	No
Lactate	+0.2	No
Pyruvate	+0.1	No
Oxaloacetate	+0	No
Acetate	-0.2	No
Succinate	-0.3	No
cis-Aconitate	Not tested	No
Isocitrate	Not tested	No
Valerate	Not tested	No
Glutarate	Not tested	No
2-Aminoadipate	Not tested	No

Table S2) Binding studies of different ligands to recombinant McpK-LBD. The upper part shows compounds that altered the McpK-LBD Tm by at least 2 °C in a thermal shift assays screen of compounds listed in Supp. Fig. 1. The last column indicates the outcome of ITC studies of these compounds to McpK-LBD. The lower part shows compounds with structural similarity to αKG that were analyzed for binding to McpK-LBD by ITC.
